# The complete mitochondrial genome of *Euthrix laeta* (Lepidoptera: Lasiocampidae)

**DOI:** 10.1080/23802359.2016.1197065

**Published:** 2016-07-23

**Authors:** Liuyu Wu, Xuming Wu, Xiao Xiong, Tianrong Xin, Yayu Wang, Zhiwen Zou, Bin Xia

**Affiliations:** a College of Life Science, Nanchang University, Nanchang, China;; b Plant Protection Station, County of Dayu, Ganzhou, China

**Keywords:** Euthrix laeta, mitochondrial genome, phylogeny

## Abstract

The complete mitochondrial genome of *Euthrix laeta* (GenBank accession number KU870700) was sequenced by traditional PCR amplification and primer walking methods. The total length was 15,368 bp, including 13 protein-coding genes, 2 ribosomal RNA genes, 22 transfer RNA genes and a A + T-rich region. The base composition of the genome was A (40.85%), T (39.34%), C (12.01%) and G (7.8%), respectively. The arrangement of all genes was identical to other lepidopteran insects. The phylogenetic relationships were established based on the nucleotide sequences of 13 protein-coding genes of mitochondrial genomes by the neighbor-joining method. The molecular-based phylogeny supported the traditional morphological classification on relationships within Lepidoptera species.

*Euthrix laeta* (Walker) (Lepidoptera: Lasiocampidae) is the wide pest which damaged bamboo and caused huger economic losses every year (Zhu et al. 2010). There have been many reports about integrated control of *E. laeta* in natural reserves (Chen & Chen 2008; Hao 2012). However, little study focused on its genetic characteristics. Therefore, it is very important to obtain the complete mitochondrial genome (mitogenome) sequence of *E. Laeta* for phylogenetic analysis of Lasiocampidae species.

The samples were collected from Taohong Ridge National Sika Deer Nature Reserve (116°42'10” E, 29°47'7” N), Jiujiang City, Jiangxi Province, China. The species identification was conducted by looking up the relevant literature and books (Liu & Wu 2006). The specimen was maintained in Natural Science Museum, College of Life Science, Nanchang University, Nanchang, China, with an accession number 150726NC. The adult of *E. Laeta* were removed wings and stored in 75% ethanol at −40 °C refrigerator for further use. Total genomic DNA was extracted from single specimen by using GENERAY BIOTECH Genomic DNA kit (Shanghai, China). The traditional PCR amplification and primer walking methods (Wei et al. 2009; Cao et al. 2012) were necessary. Six short fragments were amplified based on general primers of insects (Simon et al. 2006). According to these six specific fragments, we designed the primers of long fragments and amplified all the fragments. All the PCR products were sequenced by Sangon Biotech (Shanghai, China). Then, we obtained the complete mitogenome of *E. Laeta.* Genetic code, start and stop codons and codon bias were identified by being compared with the complete mitogenome of other Lasiocampidae species by Clustal X 1.83 software (Thompson et al. 1997) that had been submitted to NCBI and the 22 tRNA genes were identified by the online software MITOS Web Server (http://mitos.bioinf.uni-leipzig.de/index.py). In this study, the complete mitogenome of *E. Laeta* was first determined 15,368 bp in length with GenBank Accession NO. KU870700.

The complete mitogenome of *E. laeta* contained 13 protein-coding genes (PCGs), 2 ribosomal RNA genes, 22 transfer RNA genes and a A + T-rich region. The arrangement of all genes was identical to other lepidopteran insects that had been regarded as a synapomorphy for Lepidoptera (Kim et al. 2010). *ND5*, *ND4*, *ND4L*, *ND1* and eight tRNA genes (*tRNA^Gln^*, *tRNA^Cys^*, *tRNA^Tyr^*, *tRNA^Phe^*, *tRNA^His^*, *tRNA^Pro^*, *tRNA^Leu^* and *tRNA^Val^*) were encoded on the light strand; the remaining genes were encoded on the heavy strand. Except for *COI* with CGA start codon, respectively, the remaining PCGs initiated with the three orthodox start codons (ATN). The abnormal start codon in the *COI* gene was found presently in all mitochondrial genomes of Lepidoptera (Lee et al. 2006). Three PCGs (*COI, COII* and *ND4*) used incomplete stop codon T, which was commonly reported in other invertebrates (Masta & Boore 2004). The other 10 PCGs stopped with complete stop codon TAA. The length of 22 tRNA genes ranged from 64 to 71 bp. *16S rRNA* was located between the *tRNA^Leu^* and *tRNA^Val^*, *12S rRNA* was located between *tRNA^Val^* and A + T-rich region on the light strand. The A + T-rich region was located between *12S rRNA* and *tRNA^Met^* with a length of 372 bp. This region was believed to be involved in the regulation of transcription and control of DNA replication (Zhang & Hewitt 1997). The overall nucleotide composition of *E. laeta* was A (40.85%), T (39.34%), C (12.01%) and G (7.80%), which showed a high A + T bias.

In addition, establishment of the phylogeny among Lepidoptera species was based on the nucleotide sequences of the 13 PCGs. As shown in Figure 1, *E. laeta* was grouped with *Dendrolimus punctatus wenshanensis* (Lepidoptera: Lasiocampidae), *Dendrolimus punctatus* (Lepidoptera: Lasiocampidae), *Dendrolimus spectabilis* (Lepidoptera: Lasiocampidae) and *Dendrolimus tabulaeformis* (Lepidoptera: Lasiocampidae) in one branch; *Manduca sexta* and *Sphinx morio* were grouped in the family of Sphingidae; *Bombyx mori*, *Bombyx huttoni*, *Rondotia menciana* and *Bombyx mandarina* were grouped in the family of Bombycidae. Then the three branches were grouped with in the family of Lepidoptera, which was consistent with the morphological classification within the Lepidoptera insects.

**Figure 1.**
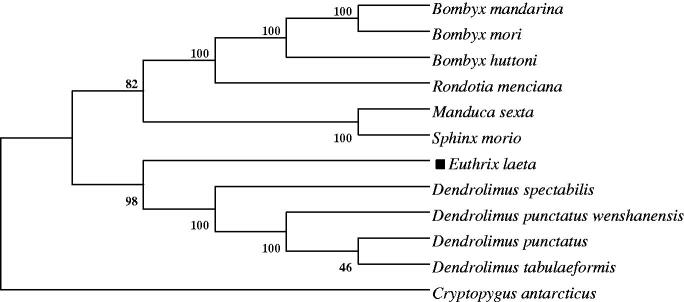
Neighbor-Joining phylogeny based on the nucleotide sequences of 13 mitochondrial PCGs using MEGA 5.05 software (Tamura et al. 2011). The Collemola species, *Cryptopygus antarcticus* used as an outgroup. The genbank accession numbers of species used in phylogenetic tree, *Bombyx mori* (AC: AB083339); *Bombyx mandarina* (AC: AB070263); *Manduca sexta* (AC: NC_010266); *Sphinx morio* (AC: NC_020780); *Bombyx huttoni* (AC: NC_026518); *Rondotia menciana* (AC: KJ647172); *Dendrolimus punctatus wenshanensis* (AC: KJ913811); *Dendrolimus punctatus* (AC: NC_027156); *Dendrolimus spectabilis* (AC: KJ913815); *Dendrolimus tabulaeformis* (AC: NC_027157); *Cryptopygus antarcticus* (AC: NC_010533).
